# Identification of Bacterial Pathogens at Genus and Species Levels through Combination of Raman Spectrometry and Deep-Learning Algorithms

**DOI:** 10.1128/spectrum.02580-22

**Published:** 2022-10-31

**Authors:** Liang Wang, Jia-Wei Tang, Fen Li, Muhammad Usman, Chang-Yu Wu, Qing-Hua Liu, Hai-Quan Kang, Wei Liu, Bing Gu

**Affiliations:** a Laboratory Medicine, Guangdong Provincial People’s Hospital, Guangdong Academy of Medical Sciences, Guangzhou, Guangdong Province, China; b Department of Intelligent Medical Engineering, School of Medical Informatics and Engineering, Xuzhou Medical University, Xuzhou, Jiangsu Province, China; c Laboratory Medicine, The Fifth People’s Hospital of Huai’an, Huai’an, Jiangsu Province, China; d Department of Biomedical Engineering, School of Medical Imaging, Xuzhou Medical University, Xuzhou, Jiangsu Province, China; e State Key Laboratory of Quality Research in Chinese Medicines, Macau University of Science and Technology, Taipa, Macau SAR, China; f Laboratory Medicine, The Affiliated Hospital of Xuzhou Medical University, Xuzhou, Jiangsu Province, China; ATCC

**Keywords:** Raman spectroscopy, SERS, bacterial pathogen, rapid diagnosis, machine learning algorithm

## Abstract

The rapid and accurate identification of the causing agents during bacterial infections would greatly improve pathogen transmission, prevention, patient care, and medical treatments in clinical settings. Although many conventional and molecular methods have been proven to be efficient and reliable, some of them suffer technical biases and limitations that require the development and application of novel and advanced techniques. Recently, due to its cost affordability, noninvasiveness, and label-free feature, Raman spectroscopy (RS) is emerging as a potential technique for fast bacterial detection. However, the method is still hampered by many technical issues, such as low signal intensity, poor reproducibility, and standard data set insufficiency, among others. Thus, it should be cautiously claimed that Raman spectroscopy could provide practical applications in real-world settings. In order to evaluate the implementation potentials of Raman spectroscopy in the identification of bacterial pathogens, we investigated 30 bacterial species belonging to 9 different bacterial genera that were isolated from clinical samples via surfaced enhanced Raman spectroscopy (SERS). A total of 17,149 SERS spectra were harvested from a Raman spectrometer and were further analyzed via machine learning approaches, which showed that a convolutional neural network (CNN) deep learning algorithm achieved the highest prediction accuracy for recognizing pathogenic bacteria at both the genus and species levels. In summary, the SERS technique holds a promising potential for fast bacterial pathogen identification in clinical laboratories with the integration of machine learning algorithms, which might be further developed and sharpened for the direct identification and prediction of bacterial pathogens from clinical samples.

**IMPORTANCE** In this study, we investigated 30 bacterial species belonging to 9 different bacterial genera that were isolated from clinical samples via surfaced enhanced Raman spectroscopy (SERS). A total of 17,149 SERS spectra were harvested from a Raman spectrometer and were further analyzed via machine learning approaches, the results of which showed that the convolutional neural network (CNN) deep learning algorithm could achieve the highest prediction accuracy for recognizing pathogenic bacteria at both the genus and species levels. Taken together, we concluded that the SERS technique held a promising potential for fast bacterial pathogen diagnosis in clinical laboratories with the integration of deep learning algorithms, which might be further developed and sharpened for the direct identification and prediction of bacterial pathogens from clinical samples.

## INTRODUCTION

Infectious diseases are a group of illnesses that are caused by microorganisms, such as viruses, parasites, bacteria, or fungi, and can spread from person to person (communicable diseases) or from an animal to a person (zoonoses) (1, 2). Although significant progress has been made by humans in fighting against infectious diseases, bacterial pathogens are still one of the major causing agents of human infections, posing great global public health threats due to infection persistence via contributing factors, such as biofilm formation and antibiotic resistance, among others (3). In addition, emerging opportunistic bacterial pathogens that do not normally infect healthy individuals could also cause infections in hospitals to immunocompromised patients or to people with underlying diseases (4), which makes it challenging to recognize these pathogens. Thus, the fast, accurate, and simplified identification of infection-causing bacterial species is key to laboratory diagnoses in clinical settings and could greatly improve patient outcomes during therapeutic treatment.

Conventionally, bacterial pathogens are mainly identified via microscopy, medium culture, and biochemical testing techniques, which are also considered the gold standard of pathogen detection and antimicrobial susceptibility testing (5). Morphological and biochemical characteristics are actually indirect comparisons of bacterial genomes, and they are more convenient than the direct analysis of bacterial genomes (6). However, in some cases, traditional methods only provide preliminary diagnostics that require further confirmations through other testing methods. For example, bacterial culture is normally undertaken under aerobic conditions in a clinical laboratory, and this largely neglects anaerobic bacterial pathogens, not even mentioning the fastidious and non-culturable bacterial species (7). As for biochemical testing, it also cannot accurately reflect the genomic complexity of a bacterium, as biochemical phenotypes are not stable and are largely dependent on metabolic states and environmental conditions (8). Thus, novel and advanced techniques have been developed for the identification of infectious diseases, such as molecular diagnostics (e.g., polymerase chain reaction [PCR]), chemical analysis (e.g., mass spectrometry [MS]), and next-generation sequencing (NGS), among others, which are able to reduce the turnaround times of test results, leading to significant impacts on clinical diagnoses (9). It is noteworthy that limitations and restrictions of the advanced diagnostic methods have also been reported. For example, although PCR is highly sensitive and specific, it cannot distinguish between viable and non-viable bacteria, and as for the immunological methods, such as enzyme-linked immunosorbent assay (ELISA), they are highly specific and have high-throughput capacity, but they require specific antibodies (10). Matrix-assisted laser desorption ionization-time-of-flight mass spectrometry (MALDI-TOF MS) is a convenient and sensitive tool for the identification of commonly encountered bacterial species in the clinical laboratory setting; however, it also suffers from several disadvantages, including an inability to discriminate related bacterial species, and it incurs high instrument costs, among other drawbacks (10, 11).

Raman spectroscopy (RS) is an emerging technique for the detection of bacterial pathogens and testing the antibiotic susceptibility of bacteria (12). Although it is too early to claim that Raman spectroscopy can be applied in routine clinical settings at the current stage due to the gaps between basic research and clinical implementation, it holds promising potential for the low cost, noninvasive and label-free detection of bacterial pathogens in laboratories (12). Thus, it is meaningful to investigate the capacity of Raman spectroscopy in the identification of different types of bacterial pathogens. So far, a variety of work has been done to identify bacterial species either simply at the single cell level or directly in clinical samples. For example, Rosch et al. reported the application of micro-Raman spectroscopy in the identification of single bacterial cells without any cultivation step, according to which the four bacterial strains Escherichia coli DSM423, Escherichia coli DSM498, Pseudomonas thermotolerans, and Pseudomonas stutzeri could be well separated (13). Strola et al. used Raman spectroscopy to study a total of 1,200 Raman spectra over 7 bacterial species, achieving a 90% accuracy of classification rate and suggesting that a Raman spectrometer could be a high-throughput method for nondestructive, real-time bacteria identification (14). In addition, Kumar et al. constructed a Raman spectroscopy protocol that could successfully differentiate dead cells from viable cells with 98% sensitivity, and this method was also able to identify viable but nonculturable cells (VBNC) that could not be recognized via conventional characterization tools (15).

Although Raman spectroscopy is promising for clinical applications, it has its own intrinsic issues, such as poor reproducibility and a low signal-to-noise ratio (SNR), among others, and these need to be addressed within the foreseeable future in order to facilitate its real-world application (16, 17). Surface enhanced Raman spectroscopy (SERS) is an effective method for solving the low SNR issue associated with conventional RS, which uses rough gold or silver metal nanoparticles to enhance SNR by many orders of magnitude (18). Thus, SERS fingerprinting spectra are then generated and further analyzed via computational methods for bacterial discrimination and prediction. Considering the sophistication of Raman spectral data, multivariate statistical methods and machine learning (ML) algorithms, rather than conventional linear analyses, are regularly used in data processing procedures (12). Supervised learning consists of a group of algorithms that are commonly used for SERS spectral analyses, such as convolutional neural networks (CNN), and support vector machines (SVM) are used for constructing spectral models and predicting sample types (19). A recent study by Ho et al. trained a CNN model to classify bacterial spectra, and this model could accurately identify 30 common bacterial pathogens (>82%) and antibiotic treatments (97.0 ± 0.3%) with low signal-to-noise ratios (20). In this study, we analyzed a large number of surface enhanced Raman spectra (*N* = 17,149) that belonged to 9 genera and 30 bacterial species that were isolated directly from clinical samples. The average SERS spectra was visualized, and the characteristic peaks for each spectrum were analyzed. A CNN deep learning algorithm was applied to pathogen analysis at the genus and species levels, and its results were compared against those of three traditional machine learning algorithms. The results showed that CNN achieved a 99.80% classification accuracy at the genus level and a 98.37% classification accuracy at the species level. The result of 5-fold cross-validation was also greater than 98%. We also used receiver operating characteristic (ROC) curves and confusion matrices to visually demonstrate model performance. In summary, our results show that although the application of Raman spectroscopy in bacterial identification is still in its infancy stage, it holds potential for the accurate clinical diagnoses of a variety of bacterial pathogens. This shall be further explored for its direct analysis of clinical samples, in terms of the discrimination of bacterial genera and species.

## RESULTS

### Average SERS spectra and characteristic peaks.

**FIG 1:**
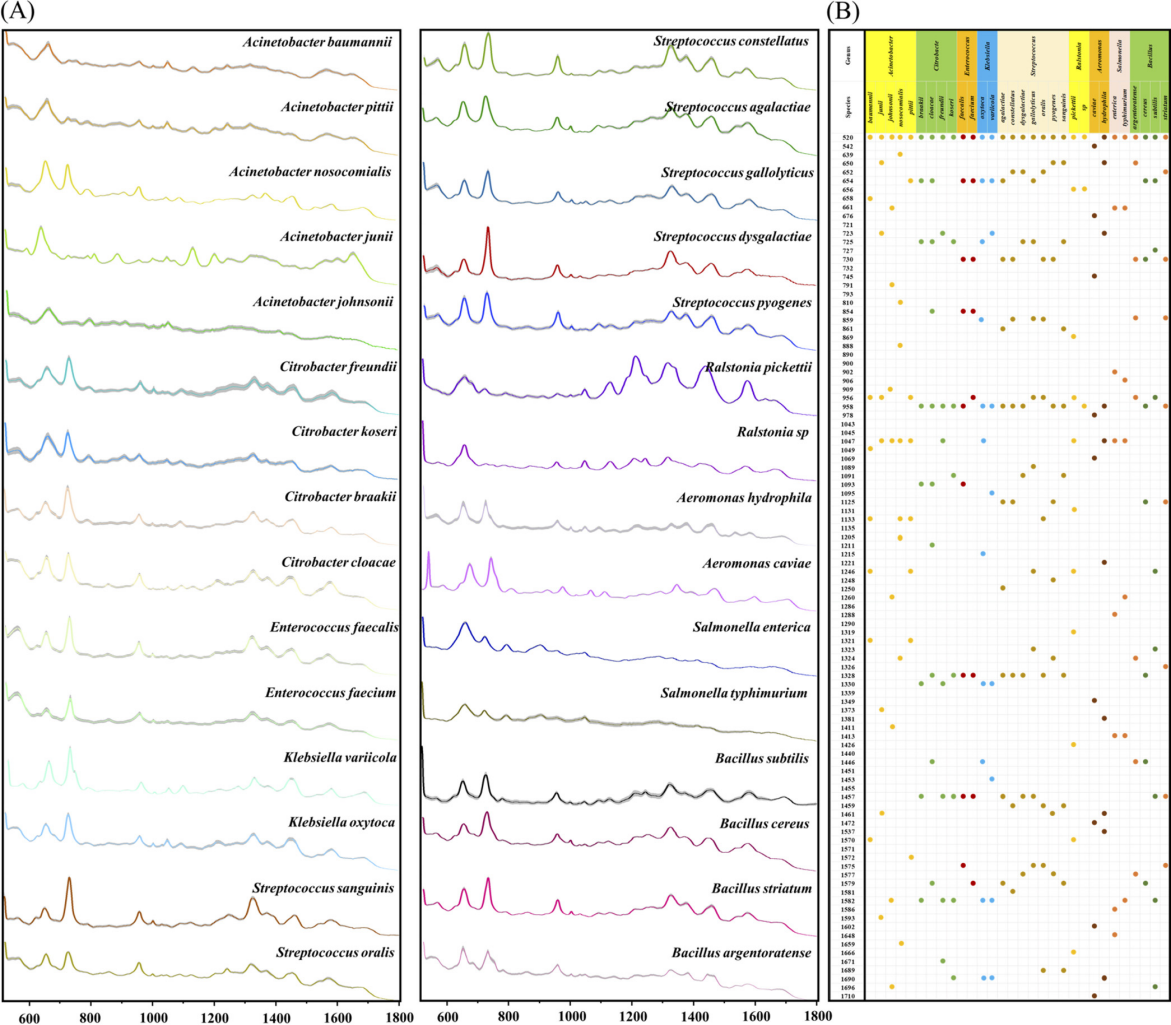
Average Raman spectra of 30 bacterial species and corresponding characteristic peaks. (A) Average SERS spectra of 30 different clinical bacterial pathogens. The shaded area represents 20% of the standard error band. (B) Dot matrix indicating the distribution of the characteristic peaks for the SERS spectra. The vertical axis represents the Raman shift, and the horizontal axis represents the different bacterial genera and species. If a pathogen has a characteristic peak at a particular Raman shift in the matrix, the corresponding location will be marked with a solid dot. The different colors of the solid dots represent different bacterial genera.

In this study, we calculated the averaged Raman intensity at each Raman shift in order to generate the averaged SERS spectrum for each of 30 bacterial species, which together belonged to 9 bacterial genera. Different bacterial species showed differences in their Raman intensities and in the distributions of their characteristic peaks, which could be used for the discrimination of these bacterial species. In addition, the standard error band was visualized in the averaged Raman spectrum of each bacterium to indicate whether the spectral data had good repeatability during SERS spectral generation. The narrower the error band, the higher the repeatability of the Raman spectrum. According to the results in Fig. 1A, the reproducibility of the Raman spectra of each pathogenic bacterium was in a good condition. Furthermore, the averaged Raman spectra of the 9 bacterial genera were also calculated and visualized, and these results are shown in Fig. S1.

Different bacterial species have unique biochemical compositions, and these were also reflected in the unique combinations of the characteristic peaks of the averaged SERS spectra. It could also be observed that the characteristic peaks of bacterial strains belonging to the same genus were relatively similar, while the characteristic peaks of bacterial strains belonging to different genera were largely different. A dot matrix diagram (Fig. 1B) describes the distributions of the characteristic peaks in all 30 of the pathogenic bacterial species. The specific characteristic peaks and their corresponding biological meanings are provided in detail in Table S2. As for the biological meanings of the characteristic peaks for the 9 bacterial genera, the specific metabolites are reported in Table S4.

### Supervised machine learning analysis.

We used a CNN deep learning algorithm to analyze the SERS spectral data generated from the 9 different bacterial genera and compared its results with those of three traditional machine learning algorithms (random forest [RF], support vector machine [SVM], and Adaboost). Four evaluation metrics, Accuracy (ACC), Precision (Pre), Recall, and F1 were applied to measure the performance of all of the machine learning models. 5-fold cross-validation was used to detect whether or not the models were overfitting during training. The results are shown in Table 1, according to which the CNN algorithm achieved the best classification accuracy (99.80%). The 5-fold cross-validation reached 98.89%, indicating that the CNN model had good stability. Excluding for the Adaboost algorithm, the RF algorithm (98.78%) and SVM algorithm (98.03%) could also achieve comparatively good classification results. In terms of the calculation efficiency, we also calculated the training time of each model, which showed that the RF model had the highest efficiency (89 s), whereas the CNN model took (97 s) for the process. As for the SVM and AdaBoost models, their calculation time exceeded (120 s). Combined with the model classification accuracy and training time, we concluded that the CNN model had the best performance for SERS spectral analysis at the level of bacterial genus.

**TABLE 1:** Comparison of the predictive abilities of four supervised machine learning algorithms at the level of bacterial genus

Algorithm	ACC	Pre	Recall	F1	5-Fold CV	Time
CNN	99.80%	98.81%	98.96%	99.19%	98.89%	97 s
RF	98.95%	98.79%	99.01%	98.97%	98.78%	89 s
SVM	98.49%	97.36%	97.56%	97.52%	98.03%	151 s
Adaboost	50.55%	50.55%	46.61%	45.63%	48.72%	149 s

Bacterial genus means closely related bacterial species with similar characteristics. As in this study, the *Enterococcus* genus contains two strains: Enterococcus faecalis and Enterococcus faecium. To confirm whether different supervised machine learning algorithms can discriminate between different pathogenic bacteria strains at the species level, we used the same method as at the genus level. The results in Table 2 show that the CNN model consistently achieved the best prediction accuracy (ACC = 98.37%), and the 5-fold cross-validation score reached 98.57%. Compared to the other three traditional machine learning algorithms in terms of calculation efficiency, the CNN model achieved the best prediction accuracy with the shortest time (94 s).

**TABLE 2:** Comparison of the predictive abilities of four supervised learning algorithms at the level of bacterial species

Algorithm	ACC	Pre	Recall	F1	5-Fold CV	Time
CNN	98.37%	98.54%	98.81%	98.61%	98.57%	94 s
RF	96.38%	96.38%	96.84%	96.97%	95.08%	147 s
SVM	97.31%	96.31%	97.14%	97.37%	97.70%	124 s
Adaboost	29.04%	29.39%	27.87%	25.90%	29.39%	227 s

**FIG 2:**
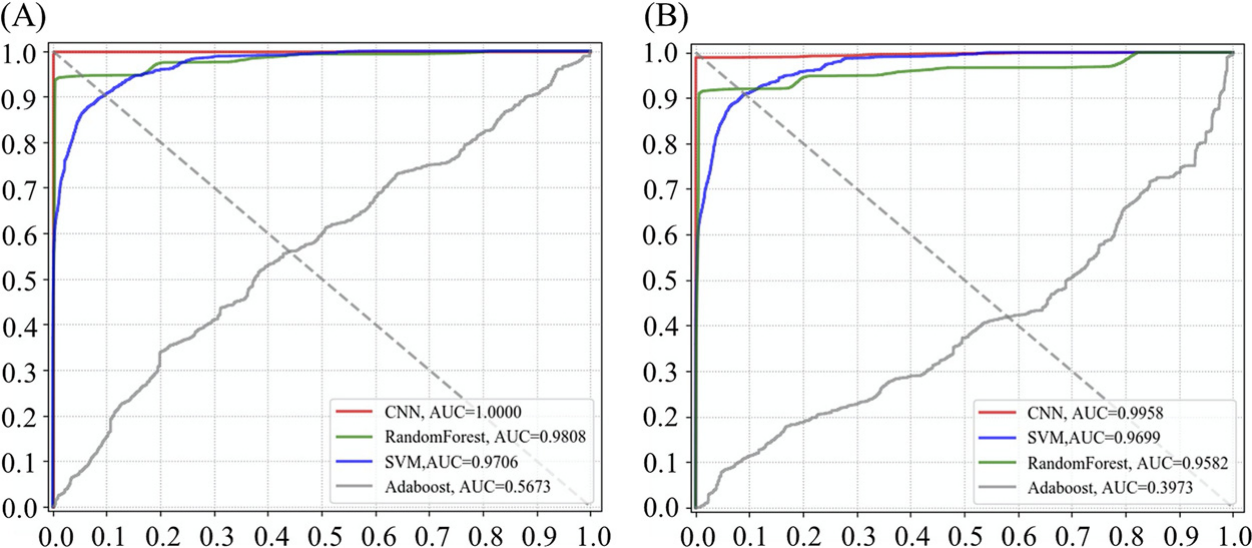
Comparison of receiver operating characteristic (ROC) curves via the area under the curve (AUC) values for four supervised machine learning algorithms at the (A) genus level and (B) species level.

To further validate the performance of the different supervised learning models on different bacterial genera and species, we used ROC curves to measure the specificity and sensitivity of each model (Fig. 2). The *x* axis of the ROC curve represents the specificity (false-positive rate, [FPR]) while the *y* axis of the curve represents the sensitivity (true positive rate, [TPR]). In the ROC curve, the closer to the upper left area, the higher the TPR and the lower the FPR of the model. Simultaneously, we also calculated the area under the curve (AUC) values of the areas under the ROC curves to measure the performances of all of the models. The larger the AUC value, the better the performance of the model. The results showed that the CNN model achieved the best performance at both the genus (AUC score = 1.000) and species levels (AUC score = 0.9958). Although the accuracies of the SVM and RF models decrease with the subdivision of the data sets, they could still achieve good AUC scores (≥0.95). However, the Adaboost model failed to correctly distinguish bacterial strains at both the genus and species levels.

### Confusion matrix analysis.

**FIG 3:**
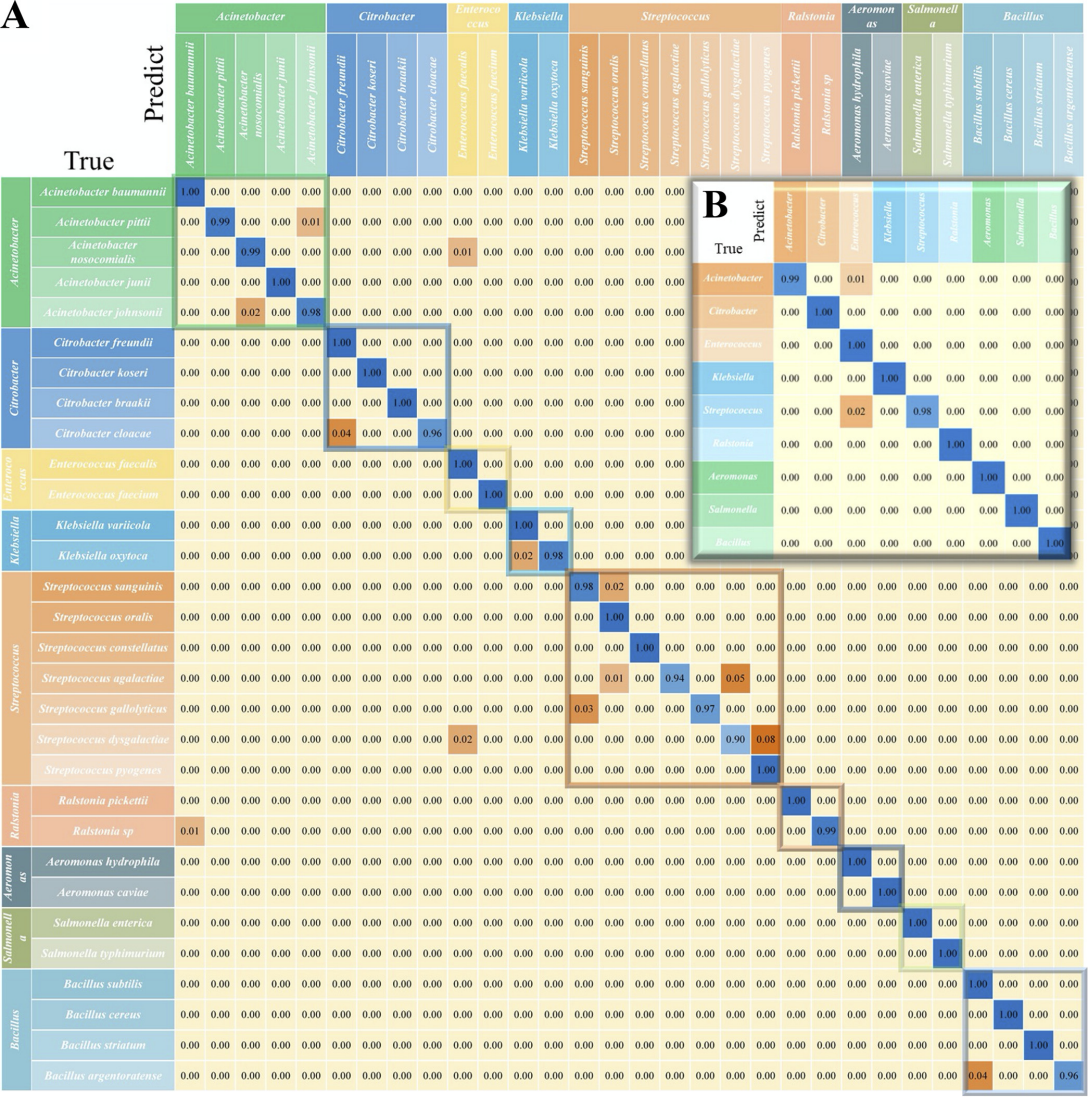
Confusion matrix of the CNN model for bacterial pathogens at the (A) genus level (9 genera) and (B) species level (30 species). Each row in the matrix represents an instance in the true class, and each column in the matrix represents an instance in the predicted class. The diagonal line represents the prediction accuracy of the CNN model on different bacterial strains. The average prediction accuracy of the CNN model was 99.67% at the bacterial genus level and 95.5% at the bacterial species level.

A confusion matrix is a quantitative visualization method used in machine learning analysis in order to summarize the prediction results of a classification model. A confusion matrix aims to describe the relationships between the real attributes of sample data and the predicted results in the form of a matrix. Therefore, it is an efficient method by which to evaluate the performance of a machine learning classifier. In this study, we chose the best-performing model (CNN) for the calculation of the confusion matrix at both the bacterial genus and species levels (Fig. 3). It can be seen from the results in Fig. 3A that, for the 30 different bacterial species, the CNN model achieved good predictive ability, and the classification accuracy of each strain was greater than or equal to 90%. Consider the genus Streptococcus as an example. Only 90% of the strain Streptococcus dysgalactiae was correctly predicted as itself, while 2% of S. dysgalactiae was misclassified as Citrobacter cloacae, and 8% of S. dysgalactiae was misclassified as Streptococcus pyogenes. In addition, 3% of Streptococcus gallolyticus was predicted to be Streptococcus sanguinis, while 2% of Streptococcus sanguinis was predicted to be Streptococcus oralis. As for Streptococcus agalactiae, 1% of the strain was predicted to be Streptococcus oralis, and 5% was predicted to be Streptococcus dysgalactiae. It is also noteworthy that different genera are enclosed in boxes of different colors. As for the confusion matrix of the bacterial genera, the CNN model could achieve a general prediction accuracy of greater than 98% in each genus, in which 1% of Acinetobacter and 2% of Streptococcus were incorrectly predicted as the genus Enterococcus (Fig. 3B).

## DISCUSSION

Pathogenic bacteria are widely distributed in hospitals, foods, and external environments, such as water and soil, which greatly increase the transmission risks and seriously endanger public health (21). Therefore, the rapid identification of pathogenic bacteria at the early stages of transmission and infection through advanced techniques could provide useful information for the prevention and treatment of bacterial infections, leading to an effective reduction of population morbidity and mortality (22). Although Raman spectroscopy has potential in the rapid detection of bacterial pathogens, it has not been applied in real clinical situations due to its technical limitations, including weak signal intensities and poor reproducibility (23). Surface enhanced Raman spectroscopy effectively overcomes the weak Raman scattering effect by combing metallic nanomaterials with Raman spectroscopy, which significantly facilitates the application potential of the technique in clinical settings (24). In spite of the good signal intensity and data-reproducible quality of the SERS technique, it still suffers from the negative influences of external factors during data acquisition, such as cosmic noise, the fluorescence background, mechanical vibration, and artificial factors. In addition, due to the sophistication of Raman spectral data, classical statistical methods are not sufficient for the spectral data analysis (25). Thus, the preprocessing procedures of the raw SERS spectral data, together with further machine learning analysis of the SERS spectra, is indispensable (26). Wang et al. recently summarized the applications of Raman spectroscopy in bacterial infections with a focus on the advantages and shortcomings of the technique, thereby highlighting the promising potential of the technique as well as the current challenges precluding its real-world applications (12).

Many previous studies have explored the possibilities of combining the SERS technique with machine learning algorithms in the rapid detection of bacterial pathogens (12, 27–29). For example, Ciloglu et al. performed rapid identification of methicillin-resistant and methicillin-sensitive Gram-positive Staphylococcus aureus strains by using supervised classification algorithms, according to which the k-nearest neighbors (KNN) classification algorithm showed the best classification accuracy (97.8%), indicating that SERS combined with machine learning is capable of discriminating antibiotic-resistant and susceptible bacteria and is promising for clinical applications (30). However, little work on deep learning algorithms was tested in the SERS spectral analysis until recently, when Ho et al. adapted the CNN deep learning algorithm from image analysis to a low signal-to-noise ratio and one-dimensional SERS spectral data, which showed that the prediction accuracies of 30 common bacterial pathogens exceeded 82% and that the identification accuracy of antibiotic treatments reached 97.0 ± 0.3% (20). In order to explore the superiority of deep learning algorithms to classical machine learning algorithms in terms of the analysis of bacterial SERS spectra, Tang et al. collected 2,752 SERS spectra belonging to 9 Staphylococcus species and compared the prediction accuracy of the representative CNN deep learning algorithm with 8 classical machine learning algorithms, including KNN and the support vector machine, among others, and these results revealed that the CNN performed consistently better than any other classical machine learning algorithm, based on all of the evaluation metrics (31). As for SERS-assisted antibiotic resistance analysis in Klebsiella pneumoniae, Liu et al. also confirmed that the CNN deep learning algorithm performed the best (5-fold cross-validation accuracy = 99.78%) compared with all of the other machine learning algorithms (23). Recently, Tang et al. investigated the effects of machine learning algorithms on 15 bacterial species, which also showed that the CNN could achieve as high as a 99.86% prediction accuracy with the highest area (0.9996) under the receiver operating characteristic curve (32). It is interesting to note that another deep learning algorithm, namely long short-term memory (LSTM), did not perform as well as did CNN, and, in some cases, it was not even as good as the classical machine learning algorithms, such as the random forest and KNN (31, 32). However, a recent study by Yu et al. showed that the LSTM method was actually faster and more accurate than was the normal CNN model, achieving an average isolation-level accuracy of greater than 94%, which requires further exploration (33).

In this study, we aimed to reveal the intrinsic differences among the surface enhanced Raman spectra of 30 bacterial species belonging to 9 different genera which harbored higher numbers of bacterial species than those used in previous studies. In addition, the prediction abilities of four supervised machine learning algorithms (1 CNN deep learning algorithm and 3 classical machine learning algorithms: RF, SVM, and Adaboost) were thoroughly compared. All of the bacterial species were isolated directly from clinical samples through medium culture. Through comparisons of the evaluation indicators (ACC, Pre, Recall, F1, 5-Fold CV, and Time) for all of the machine learning algorithms, it is clear to see in Table 1 that the performance of CNN algorithm was similar to that of the RF algorithm at genus level, and both of these had better performances than did the other two algorithms. Also, in Table 2, the performance of CNN was consistently better than all of the other classical machine learning algorithms at the species level. The results indicated that when the data complexity increased, the CNN deep learning algorithm displayed better robustness than did the classical machine learning algorithms, which is consistent with previous reports (12, 20, 23, 33). In particular, 5-fold cross-validation was used to verify that the CNN model scored with a prediction accuracy of 98.89% at the genus level and 98.57% at the species level. In addition, we also showed, for the first time, that the training times of different models were different and that the CNN algorithm had the fastest speed for bacterial prediction, taking only 97 s and 94 s to reach the best classification accuracy at the genus and species levels, respectively. Therefore, the CNN deep learning algorithm can classify and predict bacterial SERS spectra with both high accuracy and high computational efficiency (12).

### Conclusion.

The application potential of SERS techniques in the rapid analysis of pathogenic bacteria has been extensively studied from bacterial differentiation to antibiotics resistance profiling. However, real-world applications of the SERS technique are still limited and require further investigations. In this study, we recruited SERS to detect bacterial pathogens at both the genus (9 genera) and species (30 species) levels with the assistance of machine learning algorithms. The results showed that the CNN deep learning algorithm achieved the best classification and predication capacities, as quantified through different evaluation indices, such as Accuracy, Precision, F1-score and 5-fold cross validation, among others. However, all of the bacterial pathogens used in this study were dependent on clinical isolation and medium culture, which extended the turnaround time and also limited the further application of the technique in a clinical environment. In future studies, we shall apply SERS techniques to a more sophisticated real-world situation, in which bacterial pathogens might be discriminated and predicted directly from the analysis of clinical samples. Such an advancement could greatly reduce the gaps between basic research and the clinical implementation of the SERS technique. Taken together, our study confirms the potential of the integration of SERS techniques and machine learning algorithms in the rapid and accurate identification of different bacterial pathogens at both the genus and species levels, which could contribute to the progress of the prevention and treatment of bacterial pathogens in clinical settings in the near future.

## MATERIALS AND METHODS

### Chemical and biological materials.

30 clinical pathogenic bacteria belonging to 9 different bacterial genera were collected from clinical samples. A total of 17,149 SERS fingerprints were obtained for all of the bacterial species, and these are described in detail below: Acinetobacter (5 species, 82 strains, *N* = 2,490), Citrobacter (4 species, 28 strains, *N* = 2,270), Enterococcus (2 species, 47 strains, *N* = 1,255), Klebsiella (2 species, 9 strains, *N* = 1,200), Streptococcus (7 species, 35 strains, *N* = 3,985), Ralstonia (2 species, 3 strains, *N* = 1,200), Aeromonas (2 species, 7 strains, *N* = 1,200), Salmonella (2 species, 2 strains, *N* = 1,200), and Bacillus (4 species, 15 strains, *N* = 2,349). *N* represents the number of SERS fingerprinting spectra. For details regarding the bacterial species, please refer to Table S1. All of the pathogens were stored in the Department of Laboratory Medicine, the Affiliated Hospital of Xuzhou Medical University, Xuzhou, Jiangsu, China, after clinical isolation, and these were identified using biochemical methods plus MALDI-TOF MS and stored in a ThermoFisher freezer at −80°C. During bacterial culture, the colonies were taken out of a cryogenic vial (1.5 mL, Thermo Scientific Nalgene) via the sterilized inoculation loops and were then streaked on Mueller-Hinton agar plates (Sigma-Aldrich, USA), accordingly. All of the plates were incubated at 37°C for 24 h, and emerging colonies were randomly selected and mixed with a silver nanoparticle substrate for SERS analysis.

### Preparation of silver nanoparticle substrate.

33.72 mg of silver nitrate (AgNO_3_) (SinoPharm, Shanghai, China) was added to a clean triangular flask that was filled with 200 mL of deionized distilled water (ddH_2_O) and placed on a magnetic stirring heating plate (model type: ZNCl-BS230, Kesheng Pty., Ltd., Henan, China) until boiling. 8 mL of sodium citrate (Na_3_C_6_H_5_O_7_) (Sinopharm, Shanghai, China) was then added to the solution with a stirring rate of 650 rpm. Heating was stopped after 40 min, and stirring was continued until the solution cooled down to room temperature (RT). The solution was filled up to 200 mL with ddH_2_O and mixed well. 1 mL of the above solution was transferred into a clean Eppendorf (EP) tube and centrifuged at 7,000 rpm for 7 min. The supernatant was discarded, and the pellet was resuspended with 100 μL of ddH_2_O after centrifugation. A homogeneous gray solution was finally obtained and stored away from light at room temperature for long-term use.

### Surface-enhanced Raman spectroscopy and parameter setting.

After culturing each strain on agar plates overnight, single colonies were picked up, mixed with 15 μL of phosphate buffered saline (PBS), and vortexed vigorously, and these were then mixed with 15 μL of a negatively charged silver nanoparticle substrate solution. The well-mixed solution was pipetted onto a silicon wafer and left on a sterile biosafety cabinet to completely air dry, and it was then measured for SERS spectra. The commercial Raman spectrometer i-Raman 205 Plus BWS 465-785H (B&W Tek, USA) was used to measure all of the samples and generate the SERS spectra. During pathogen detection via a SERS spectrometer, a set of parameters were set, which included: (i) laser power, 20 mW; (ii) nominal laser port wavelength, 785 nm; (iii) detector type, charge coupled device (CCD) array with high quantum efficiency; (iv) Raman shift, 65 to 2,800 cm^−1^; (v) spectrum integration time, 5 s; and (vi) resolution, <3.5 cm^−1^ at 912 nm.

### Data preprocessing of SERS spectra.

In order to improve the identification accuracies of bacterial pathogens through SERS spectral data analysis and reduce the influence of noise on machine learning algorithms, it is necessary to preprocess the raw SERS spectral data. Common preprocessing methods include polynomial fitting, baseline correction, and normalization, among others. In this experiment, the software Unscrambler X (CAMO, USA, version 10.4, 64-bit) was used to smooth the SERS spectra through the Savitzky-Golay algorithm. All of the preprocessing steps were implemented by using the *Transform* function under the *Tasks* menu bar. The specific steps are as follows. First, select *Savitzky-Golay Smoothing* (Polynomial Order = 6, Number of Left/Right Side Point = 3) under *Smoothing* functions to smooth the Raman spectrum. In order to eliminate the unpredictable noise interference that is composed of flicker noise, thermal noise, and cosmic noise, the *Baseline* (Method = Baseline offset) function was used to eliminate continuous distortion and substrate-related Raman signals caused by uncontrollable factors during acquisition. Finally, the function *Normalize* (Type = maximum normalization) was used to control the differences between the Raman signal intensity levels, and the data were mapped to the range of [0, 1] for processing. All of the Raman spectra were analyzed between a range of Raman shifts of 519.56 to 1,800.81 cm^−1^.

### Averaged SERS spectra and characteristic peaks.

After the preprocessing of the SERS spectral data, the averaged SERS spectrum for each bacterial species was generated via the calculation of the intensities at each Raman shift, while the standard deviations (shaded error bands) were also generated, correspondingly (Fig. 1A). The averaged SERS spectra were then imported into the LabSpec software (HORIBA Scientific, Japan), and the *Gauss-Loren Function* was recruited to find the characteristic peaks in each averaged SERS spectrum by using the *Search* operation. The parameters were set as level (%) = 9, size (pnt) = 30, and iteration = 5. We then used a dot matrix to display the distributions of the characteristic peaks for each spectrum (Fig. 1B) and specified the biological meanings of the characteristic peaks for the 30 bacterial species by referring to the literature (Table S2).

### Supervised machine learning analysis.

In order to qualitatively identify different bacterial pathogens through SERS spectra and establish an optimal automatic classification model, it was necessary to compare the effects of different supervised machine learning algorithms. In this study, we used the convolutional neural network (CNN) deep learning algorithm to classify and predict the SERS spectra data of 9 different bacterial genera, which together contained 30 different pathogenic bacterial species. We then compared the results with those obtained by using three traditional machine learning algorithms, namely, random forest, support vector machines, and AdaBoost. Before conducting any supervised machine learning analysis, specific labels were assigned to allof the bacterial pathogens, such as “baumannii” for Acinetobacter baumannii, “hydrophila” for Aeromonas hydrophila, and “pyogenes” for Streptococcus pyogenes. Then, we used the *train_test_split* function to split all of the strain Raman spectral data into training, validation, and test sets in a ratio of 6:2:2. The test set does not participate in the training and verification of the model and is only used to detect the classification and prediction performance of the model, independently. In order to facilitate the calculation, we use the *LabelEncoder* function to convert the sample labels into continuous numerical variables. This facilitated the utilization and identification of the machine learning algorithms. During the SERS spectral data analyses for the three traditional machine learning algorithms, we preset the parameter ranges for each algorithm, searching for the best parameters through the *GridSearch* function (Table S3). 5-fold cross-validation (CV) was then applied to optimize the results. As an end-to-end training method, the CNN algorithm does not require a complex parameter setting process. In this study, the Keras deep learning development environment was used to build our CNN network structure. When analyzing different strains at the genus level, the CNN network structure mainly included an input layer, 6 convolution layers, 3 maxpooling layers, and a fully connected layer. The *input_shape* size of the input layer was (657, 1), while the size of the convolution kernel was 3×1. To improve the expressiveness of the network, each convolution layer was equipped with a *relu* activation function. Every two convolution layers were followed by a maxpooling layer so that the network could fully learn the local and global features of the samples. The parameter *pooling_size* was set to 3. In order to differentiate the SERS spectral data, a fully connected layer (*Dense*) was introduced, while the *Flatten* function was used to stretch the data into a column to promote the orderly connection of neurons. The *softmax* function was used to classify the SERS spectral data, and the *units* parameter of the Dense layer is 9. The loss function was selected as *categorical_crossentropy*, and the optimizer was selected as *adam*. When analyzing the SERS spectral data of the 30 different bacterial species, the *units* parameter was set to 30.

### Comparative evaluation of machine learning algorithms.

Metrics are essential to evaluate the effects of machine learning algorithms during data analysis. In this study, different evaluation metrics were used to measure the performance of all of the machine learning models. Accuracy (ACC) and Loss are the most commonly used performance indicators in spectrum signal recognition. However, during spectral data collection, each sample size cannot be guaranteed to be balanced. For example, the number of bacterial species contained in each genus in this study was different. That is, the genus Streptococcus contained seven strains, while the genus Klebsiella contained two strains. Data sets with small sample sizes could be computationally affected by data sets with large sample sizes. Therefore, other performance measures are needed to assess the qualities of machine learning models. When solving the problem of unbalanced binary data, the commonly used evaluation indicators are Precision (P) and Recall (R). The multiclassification problem in this study could be transformed into multiple binary classification problems. When the model was used to analyze the data set, it could be divided into one of four cases: true positive (TP), false-positive (FP), true negative (TN), and false-negative (FN). The precision rate and recall rate could then be expressed as follows.

$$P=\frac{\mathrm{TP}}{\mathrm{TP}+\mathrm{FP}},R=\frac{\mathrm{TP}}{\mathrm{TP}+\mathrm{FN}}$$

Since Precision and Recall are a pair of contradictory quantities, when P is high, R is often relatively low, and when R is high, P is often relatively low. Therefore, in order to better evaluate the performance of the classifier, the F1-score (F1) is generally used as a standard to measure the comprehensive performance of the machine learning classifier.

$$\frac{1}{F1}=\frac{1}{2}\left(\frac{1}{P}+\frac{1}{R}\right)\to F1=\frac{2\times P\times R}{P+R}$$

The larger the value of F1, the better the model performance is generally considered to be. To avoid overfitting the model, 5-fold cross-validation was used on the training data. In addition, we also demonstrated the efficiency of the model by comparing the time consumed by the model to achieve the optimal classification result. In addition to quantitative evaluation metrics, we also used receiver operating characteristic curves to visualize model performance. The value of area under the curve (AUC) was calculated by using the formula below.

$$\mathrm{AUC}=\frac{1}{2}\sum_{i=1}^{n-1}\left(\mathrm{TP}R_{i+1}+\mathrm{TP}R_{i}\right)\left(\mathrm{FP}R_{i+1}-\mathrm{FP}R_{i}\right)$$

Here, *n* represents the total number of points in the ROC curve. The (FPR_n_, TPR_n_) terms represent the coordinates of the last points of the ROC curves. In addition, the confusion matrix of the 9 bacterial genera and 30 bacterial species was also calculated and was visualized in order to check the prediction effects of the bacterial pathogens by the CNN model, which is considered to be a convenient method for the subsequent adjustment of the model.
