# Supplementary material for: Identification of Bacterial Pathogens at Genus and Species Levels through Combination of Raman Spectrometry and Deep-Learning Algorithms
Source: Microbiol Spectr. 2022 Oct 31;10(6):e02580-22. doi: 10.1128/spectrum.02580-22 (PMC9769533; doi:10.1128/spectrum.02580-22)
Supplement: Supplemental file 1 — Supplemental material. Download spectrum.02580-22-s0001.pdf, PDF file, 0.6 MB [file spectrum.02580-22-s0001.pdf]

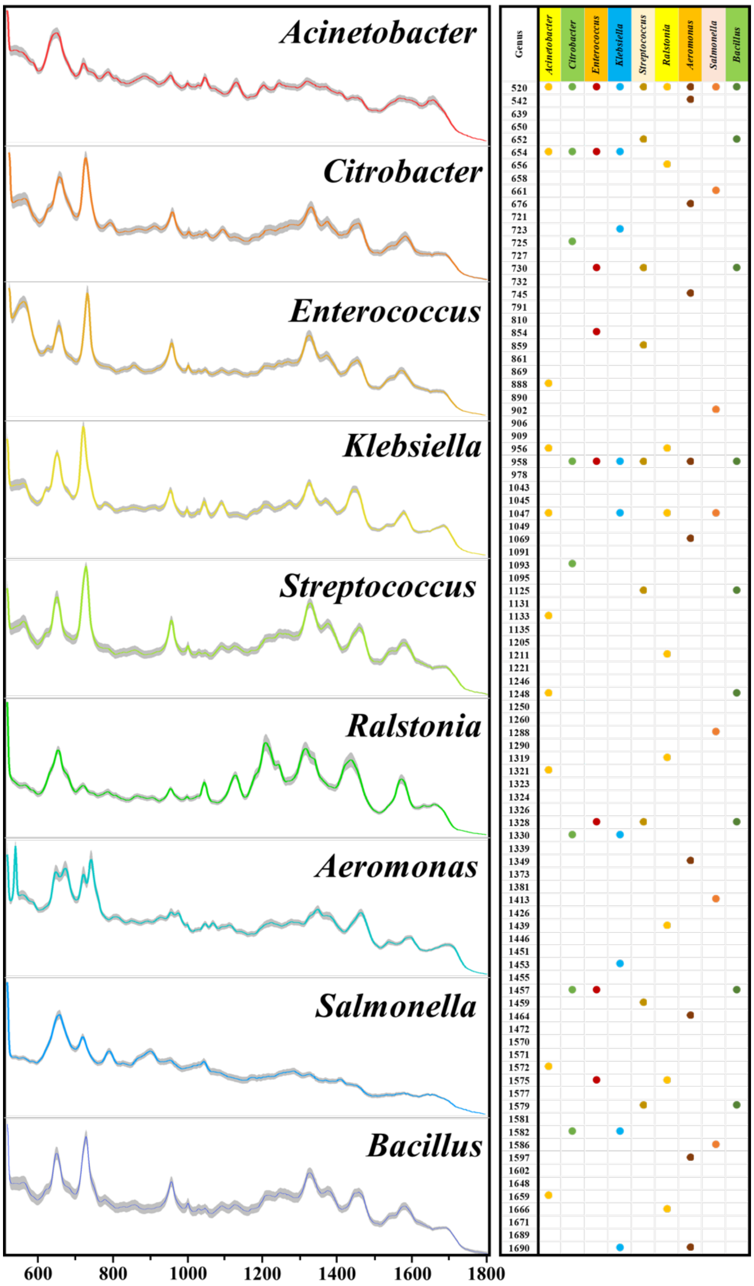

Supplementary Figure S1 Average Raman spectra of 9 bacterial genera and corresponding characteristic peaks. (A) Average SERS spectra of 9 different bacterial genera. The shaded area represents 20% of the standard error band. (B) Dot matrix indicating the distribution of characteristic peaks for SERS spectra. The vertical axis represents Raman shift while the horizontal axis represents different bacterial genera and species. If a bacterial genus has a characteristic peak at a particular Raman shift in the matrix, the corresponding location will be marked with a solid dot.

Supplementary Table S1 Genus and species of all the bacterial pathogens used in this study. The number of bacterial strains for each species and the number of SERS spectra for each species were listed.

Genus	Species	Full Binomial Names	No. of Strains	No. of Spectra
Acinetobacter	*baumannii*	*Acinetobacter baumannii*	57	280
	pittii	*Acinetobacter pittii*	11	610
	nosocomialis	*Acinetobacter nosocomialis*	6	640
	junii	*Acinetobacter junii*	3	350
	johnsonii	*Acinetobacter johnsonii*	5	610
Aeromonas	*hydrophila*	*Aeromonas hydrophila*	5	600
	caviae	*Aeromonas caviae*	2	600
Bacillus	*subtilis*	*Bacillus subtilis*	4	600
	cereus	*Bacillus cereus*	4	600
	striatum	*Bacillus striatum*	4	600
	argentoratense	*Bacillus argentoratense*	3	549
Citrobacter	*freundii*	*Citrobacter freundii*	11	700
	koseri	*Citrobacter koseri*	5	600
	braakii	*Citrobacter braakii*	8	630
	cloacae	*Citrobacter cloacae*	4	340
Enterococcus	*faecalis*	*Enterococcus faecalis*	22	655
	faecium	*Enterococcus faecium*	25	600
Klebsiella	*variicola*	*Klebsiella variicola*	2	600
	oxytoca	*Klebsiella oxytoca*	7	600
Ralstonia	*pickettii*	*Ralstonia pickettii*	2	600

	sp	*Ralstonia sp*	1	600
Salmonella	*enterica*	*Salmonella enterica*	4	600
	typhimurium	*Salmonella typhimurium*	4	600
Streptococcus	*sanguinis*	*Streptococcus sanguinis*	5	600
	oralis	*Streptococcus oralis*	9	385
	constellatus	*Streptococcus constellatus*	5	600
	agalactiae	*Streptococcus agalactiae*	8	600
	gallolyticus	*Streptococcus gallolyticus*	4	600
	dysgalactiae	*Streptococcus dysgalactiae*	2	600
	pyogenes	*Streptococcus pyogenes*	2	600

Supplementary Table S2 The biological meaning of the characteristic peaks for 30 bacterial species by referring to the literature.

Raman Shift (cm^{-1})	Band Assignment				
	Proteins	Carbohydrates	Nucleic acids	Lipids	Refs.
520	Skeletal mode				(1)
542	Amino acid cysteine				(2)
639	C-S stretch, tyrosine C-C twist				(3)
650/652/654			Guanine		(4)
656/658			Adenine		(5)
661	C-S stretch mode of cystine (collagen type I)				(6)
676			Ring breathing mode of the DNA		(3)
723/725/727/730	C-S		Adenine		(7)
745			Thymine ring breathing mode		(8)
791			O-P-O phosphodiester bands in DNA		(9)
810			O-P-O stretch in RNA		(9)
854/859	Tyrosine				(10)
861/869	Proline				(7)
888		Methylene rocking			(11)
902/906/909					
956/958	C-N stretch				(12)
978	C-C stretch			=CH bending	(9)
1047/1049		Carbohydrates			(12)
1069		Carbohydrates		C-O stretch	(4, 13)

1089/1091/1093/1095		Vibrations of C-C, C-O and C-O-H			(14)
1125				*ν*(C-C) skeletal of acyl backbone	(15)
1131/1133	C-N stretch				(3)
1205	Amide III				(3)
1211/1215	Stretch of C-N				(16)
1221	Amide III	*ν*(C-O), *ν*(C-C)			(8, 10)
1246/1248/1250	Amide III (of collagen)				(17)
1260	Amide III				(18)
1288	δ(CH2), Amide III				(6)
1319	C-H deformation		Guanine		(3, 19)
1321/1323/1324/1326/1328/1330	(C–N) stretch		DNA， *ν*(NH2) Adenine, Polyadenine		(20, 21)
1349	(C-H) deformation				(9)
1373/1381			Ring breathing modes of the DNA/RNA bases		(3)
1411/1413		Stretch C=C in the quinoid ring			(22)
1426		C–O vibrations of peptidoglycan			(14)
1446			CH_2 deformation in the deoxyribose moiety		(19)
1453/1457/1459/1461	δ (CH_2)				(21, 23)
1472			Guanine, Adenine, (C-H) deformation		(9)
1537			Cytosine		(19)

1570/1572/1575/1577/1579			Guanine, Adenine		(19)
1581/1582/1586	Phenylalanine, Amide II			C=C	(24-26)
1593/1602		C-N and C=C stretch in quinoid ring			(22)
1648/1659	C=O stretch (amide I)				(3)
1666	Collagen				(27)
1671	Amide I				(6)
1689/1690/1696	Amide I				(6)
1710		C=O			(28)

Reference

1. Liu Y, Zhou H, Hu Z, Yu G, Yang D, Zhao J. Label and label-free based surface-enhanced Raman scattering for pathogen bacteria detection: A review. Biosens Bioelectron. 2017;94:131-40.
2. Zhang S, Tang X, Zheng H, Wang D, Xie Z, Ding W, et al. Combination of bacitracin-based flocculant and surface enhanced Raman scattering labels for flocculation, identification and sterilization of multiple bacteria in water treatment. J Hazard Mater. 2021;407:124389.
3. Chan JW, Taylor DS, Zwerdling T, Lane SM, Ihara K, Huser T. Micro-Raman Spectroscopy Detects Individual Neoplastic and Normal Hematopoietic Cells. Biophysical Journal. 2006;90(2):648-56.
4. ALmagedi MAS, Yao W. SERS signatures of foodborne pathogenic zoonotic bacteria using gold colloid. International Journal of Engineering Science and Technology. 2013;5(4):810.
5. Walter A, März A, Schumacher W, Rösch P, Popp J. Towards a fast, high specific and reliable discrimination of bacteria on strain level by means of SERS in a microfluidic device. Lab Chip. 2011;11(6):1013-21.
6. Movasaghi Z, Rehman S, Rehman IU. Raman spectroscopy of biological tissues. Applied Spectroscopy Reviews. 2007;42(5):493-541.
7. Stone N, Kendall C, Smith J, Crow P, Barr H. Raman spectroscopy for identification of epithelial cancers. Faraday discussions. 2004;126:141-57.
8. Lin Z, Zhao X, Huang J, Liu W, Zheng Y, Yang X, et al. Rapid screening of colistin-resistant Escherichia coli, Acinetobacter baumannii and Pseudomonas aeruginosa by the use of Raman spectroscopy and hierarchical cluster analysis. Analyst. 2019;144(8):2803-10.
9. Notingher I, Green C, Dyer C, Perkins E, Hopkins N, Lindsay C, et al. Discrimination between ricin and sulphur mustard toxicity in vitro using Raman spectroscopy. Journal of the Royal Society Interface. 2004;1(1):79-90.

10. Ushakumari L, Varghese HT, Panicker CY, Ertan T, Yildiz I. Vibrational spectroscopic studies and DFT calculations of 4-fluoro-N-(2-hydroxy-4-nitrophenyl) benzamide. Journal of Raman Spectroscopy: An International Journal for Original Work in all Aspects of Raman Spectroscopy, Including Higher Order Processes, and also Brillouin and Rayleigh Scattering. 2008;39(12):1832-9.
11. Katainen E, Elomaa M, Laakkonen UM, Sippola E, Niemelä P, Suhonen J, et al. Quantification of the amphetamine content in seized street samples by Raman spectroscopy. Journal of forensic sciences. 2007;52(1):88-92.
12. Sengupta A, Laucks ML, Davis EJ. Surface-enhanced Raman spectroscopy of bacteria and pollen. Applied spectroscopy. 2005;59(8):1016-23.
13. Schuster KC, Urlaub E, Gapes J. Single-cell analysis of bacteria by Raman microscopy: spectral information on the chemical composition of cells and on the heterogeneity in a culture. Journal of Microbiological Methods. 2000;42(1):29-38.
14. Teixeira AM, Nemec A, Sousa C. Differentiation of Taxonomically Closely Related Species of the Genus Acinetobacter Using Raman Spectroscopy and Chemometrics. Molecules. 2019;24(1).
15. Cheng WT, Liu MT, Liu HN, Lin SY. Micro-Raman spectroscopy used to identify and grade human skin pilomatrixoma. Microsc Res Tech. 2005;68(2):75-9.
16. Naumann D, editor Infrared and NIR Raman spectroscopy in medical microbiology. Infrared spectroscopy: new tool in medicine; 1998: SPIE.
17. Cheng WT, Liu MT, Liu HN, Lin SY. Micro-Raman spectroscopy used to identify and grade human skin pilomatrixoma. Microscopy research and technique. 2005;68(2):75-9.
18. Ghebremedhin M, Heitkamp R, Yesupriya S, Clay B, Crane NJ. Accurate and Rapid Differentiation of Acinetobacter baumannii Strains by Raman Spectroscopy: a Comparative Study. J Clin Microbiol. 2017;55(8):2480-90.
19. Deng H, Bloomfield VA, Benevides JM, Thomas Jr GJ. Dependence of the Raman signature of genomic B-DNA on nucleotide base sequence. Biopolymers: Original Research on Biomolecules. 1999;50(6):656-66.
20. Zeiri L, Bronk BV, Shabtai Y, Eichler J, Efrima S. Surface-enhanced Raman spectroscopy as a tool for probing specific biochemical components in bacteria. Appl Spectrosc. 2004;58(1):33-40.
21. Kahraman M, Yazici MM, Sahin F, Culha M. Convective assembly of bacteria for surface-enhanced Raman scattering. Langmuir. 2008;24(3):894-901.
22. Laska J, Widlarz J. Spectroscopic and structural characterization of low molecular weight fractions of polyaniline. Polymer. 2005;46(5):1485-95.
23. Zhou X, Hu Z, Yang D, Xie S, Jiang Z, Niessner R, et al. Bacteria Detection: From Powerful SERS to Its Advanced Compatible Techniques. Adv Sci (Weinh). 2020;7(23):2001739.
24. Nowicka AB, Czaplicka M, Szymborski T, Kamińska A. Combined negative dielectrophoresis with a flexible SERS platform as a novel strategy for rapid detection and identification of bacteria. Anal Bioanal Chem. 2021;413(7):2007-20.
25. Rippa M, Castagna R, Sagnelli D, Vestri A, Borriello G, Fusco G, et al. SERS Biosensor Based on Engineered 2D-Aperiodic Nanostructure

for In-Situ Detection of Viable Brucella Bacterium in Complex Matrix. Nanomaterials (Basel). 2021;11(4).
26. Liu Y, Chen YR, Nou X, Chao K. Potential of surface-enhanced Raman spectroscopy for the rapid identification of Escherichia coli and Listeria monocytogenes cultures on silver colloidal nanoparticles. Appl Spectrosc. 2007;61(8):824-31.
27. Kaminaka S, Yamazaki H, Ito T, Kohda E, Hamaguchi Ho. Near-infrared Raman spectroscopy of human lung tissues: possibility of molecular-level cancer diagnosis. Journal of Raman Spectroscopy. 2001;32(2):139-41.
28. Krafft C, Neudert L, Simat T, Salzer R. Near infrared Raman spectra of human brain lipids. Spectrochimica Acta Part A: Molecular and Biomolecular Spectroscopy. 2005;61(7):1529-35.

Supplementary Table S3.A Parameter settings of machine learning algorithms among different bacteria genera.

Algorithm	Parameter	Optimum Parameter
**Adaboost**	n_estimators = [80, 100, 140, 145, 150, 160, 170, 175, 180, 185]; learning_r = [0.1, 1, 0.01, 0.5]	learning_rate': 0.1; 'n_estimators': 175
**Random Forest**	n_estimators = [120, 140, 145, 150, 155, 160]; max_depth = range(1, 10); criterions = ['gini', 'entropy']	criterion='entropy'; max_depth=9; n_estimators=120
**SVM**	Cs = [0.001, 0.01, 0.1, 1, 1.5, 2, 2.5, 3, 4, 5, 10]; gammas = [0.0001, 0.0001, 0.001, 0.01, 0.1, 1];	C=1; gamma=0.1; kernel='linear'; probability=True

Supplementary Table S3.B Parameter settings of machine learning algorithms among different bacteria species.

Algorithm	Parameter	Optimum Parameter
**Adaboost**	n_estimators = [80, 100, 140, 145, 150, 160, 170, 175, 180, 185]; learning_r = [0.1, 1, 0.01, 0.5]	learning_rate=0.1; n_estimators=150
**Random Forest**	n_estimators = [120, 140, 145, 150, 155, 160]; max_depth = range(1, 10); criterions = ['gini', 'entropy']	criterion='entropy'; max_depth=9; n_estimators=155
**SVM**	Cs = [0.0001, 0.001, 0.01, 0.1, 1, 1.5, 2, 2.5, 3, 4, 5, 10]; gammas = [0.0001, 0.001, 0.01, 0.1, 1]	C=1; gamma=0.00001; kernel='linear'; probability=True

Supplementary Table S4 The biological meaning of the characteristic peaks for 9 bacterial genera by referring to the literature.

Raman Shift (cm^{-1})	Assignment				
	Proteins	Carbohydrates	Nucleic acids	Lipids	Refs.
520	Skeletal mode				(1)
542	Amino acid				(2)
652/654			Guanine		(3)
656			Adenine		(4)
661	C-S stretch mode of cystine (collagen type I)				(5)
676			Ring breathing mode of the DNA		(6)
723/725/730	C-S		Adenine		(7)
745			Thymine ring breathing mode		(8)
854/859	Tyrosine				(9)
888		Methylene rocking			(10)
902					(11)
956/958	C-N stretch				(11)
1047		Carbohydrates			
1069		Carbohydrates		C-O stretch	(3, 12)
1093		Vibrations of C-C, C-O and			(13, 14)

		C-O-H			
1125				*v*(C-C) skeletal of acyl backbone	(14)
1133	C-N stretch				(6)
1211	Stretch of C-N				(15)
1248	Amide III (of collagen)				(14)
1288	δ(CH2), Amide III				(5)
1319	C-H deformation		Guanine		(6, 16)
1321/1328/1330	(C–N) stretch		DNA， *v*(NH2) Adenine, Polyadenine		(17, 18)
1349	(C-H) deformation				(19)
1413		Stretch C=C in the quinoid ring			(20)
1439		CH2 deformation			(21)
1453/1457/1459	δ (CH_2)				(18, 22)
1464		δ CH2		Lipids	(23, 24)
1572/1575/1579			Guanine, Adenine		(16)

1582/1586	Phenylalanine, Amide II		C=C	(25-27)
1597		C=N and C55C stretching in quinoid ring		(15)
1659	C=O stretch (amide I)			(6)
1666	Collagen			(28)
1690	Amide I			(5)

Reference

1. Liu Y, Zhou H, Hu Z, Yu G, Yang D, Zhao J. Label and label-free based surface-enhanced Raman scattering for pathogen bacteria detection: A review. Biosens Bioelectron. 2017;94:131-40.
2. Zhang S, Tang X, Zheng H, Wang D, Xie Z, Ding W, et al. Combination of bacitracin-based flocculant and surface enhanced Raman scattering labels for flocculation, identification and sterilization of multiple bacteria in water treatment. J Hazard Mater. 2021;407:124389.
3. ALmagedi MAS, Yao W. SERS signatures of foodborne pathogenic zoonotic bacteria using gold colloid. International Journal of Engineering Science and Technology. 2013;5(4):810.
4. Walter A, März A, Schumacher W, Rösch P, Popp J. Towards a fast, high specific and reliable discrimination of bacteria on strain level by means of SERS in a microfluidic device. Lab Chip. 2011;11(6):1013-21.
5. Movasaghi Z, Rehman S, Rehman IU. Raman spectroscopy of biological tissues. Applied Spectroscopy Reviews. 2007;42(5):493-541.
6. Chan JW, Taylor DS, Zwerdling T, Lane SM, Ihara K, Huser T. Micro-Raman Spectroscopy Detects Individual Neoplastic and Normal Hematopoietic Cells. Biophysical Journal. 2006;90(2):648-56.
7. Stone N, Kendall C, Smith J, Crow P, Barr H. Raman spectroscopy for identification of epithelial cancers. Faraday discussions. 2004;126:141-57.
8. Lin Z, Zhao X, Huang J, Liu W, Zheng Y, Yang X, et al. Rapid screening of colistin-resistant Escherichia coli, Acinetobacter baumannii and Pseudomonas aeruginosa by the use of Raman spectroscopy and hierarchical cluster analysis. Analyst. 2019;144(8):2803-10.
9. Ushakumari L, Varghese HT, Panicker CY, Ertan T, Yildiz I. Vibrational spectroscopic studies and DFT calculations of 4-fluoro-N-(2-hydroxy-4-nitrophenyl) benzamide. Journal of Raman Spectroscopy: An International Journal for Original Work in all Aspects of Raman

Spectroscopy, Including Higher Order Processes, and also Brillouin and Rayleigh Scattering. 2008;39(12):1832-9.
10. Katainen E, Elomaa M, Laakkonen UM, Sippola E, Niemelä P, Suhonen J, et al. Quantification of the amphetamine content in seized street samples by Raman spectroscopy. Journal of forensic sciences. 2007;52(1):88-92.
11. Sengupta A, Laucks ML, Davis EJ. Surface-enhanced Raman spectroscopy of bacteria and pollen. Applied spectroscopy. 2005;59(8):1016-23.
12. Schuster KC, Urlaub E, Gapes J. Single-cell analysis of bacteria by Raman microscopy: spectral information on the chemical composition of cells and on the heterogeneity in a culture. Journal of Microbiological Methods. 2000;42(1):29-38.
13. Teixeira AM, Nemec A, Sousa C. Differentiation of Taxonomically Closely Related Species of the Genus Acinetobacter Using Raman Spectroscopy and Chemometrics. Molecules. 2019;24(1).
14. Cheng WT, Liu MT, Liu HN, Lin SY. Micro-Raman spectroscopy used to identify and grade human skin pilomatrixoma. Microscopy research and technique. 2005;68(2):75-9.
15. Naumann D, editor Infrared and NIR Raman spectroscopy in medical microbiology. Infrared spectroscopy: new tool in medicine; 1998: SPIE.
16. Deng H, Bloomfield VA, Benevides JM, Thomas Jr GJ. Dependence of the Raman signature of genomic B-DNA on nucleotide base sequence. Biopolymers: Original Research on Biomolecules. 1999;50(6):656-66.
17. Zeiri L, Bronk BV, Shabtai Y, Eichler J, Efrima S. Surface-enhanced Raman spectroscopy as a tool for probing specific biochemical components in bacteria. Appl Spectrosc. 2004;58(1):33-40.
18. Kahraman M, Yazici MM, Sahin F, Culha M. Convective assembly of bacteria for surface-enhanced Raman scattering. Langmuir. 2008;24(3):894-901.
19. Notingher I, Green C, Dyer C, Perkins E, Hopkins N, Lindsay C, et al. Discrimination between ricin and sulphur mustard toxicity in vitro using Raman spectroscopy. Journal of the Royal Society Interface. 2004;1(1):79-90.
20. Laska J, Widlarz J. Spectroscopic and structural characterization of low molecular weight fractions of polyaniline. Polymer. 2005;46(5):1485-95.
21. Frank CJ, McCreery RL, Redd DC. Raman spectroscopy of normal and diseased human breast tissues. Analytical chemistry. 1995;67(5):777-83.
22. Zhou X, Hu Z, Yang D, Xie S, Jiang Z, Niessner R, et al. Bacteria Detection: From Powerful SERS to Its Advanced Compatible Techniques. Adv Sci (Weinh). 2020;7(23):2001739.
23. Weng S, Ling X, Song Y, Xu Y, Li W, Zhang X, et al. FTIR fiber optics and FT-Raman spectroscopic studies for the diagnosis of cancer. American Clinical Laboratory. 2000;19(7):20-.
24. Dukor RK. Vibrational spectroscopy in the detection of cancer. Handbook of vibrational spectroscopy. 2006.
25. Nowicka AB, Czaplicka M, Szymborski T, Kamińska A. Combined negative dielectrophoresis with a flexible SERS platform as a novel strategy for rapid detection and identification of bacteria. Anal Bioanal Chem. 2021;413(7):2007-20.

26. Rippa M, Castagna R, Sagnelli D, Vestri A, Borriello G, Fusco G, et al. SERS Biosensor Based on Engineered 2D-Aperiodic Nanostructure for In-Situ Detection of Viable Brucella Bacterium in Complex Matrix. Nanomaterials (Basel). 2021;11(4).
27. Liu Y, Chen YR, Nou X, Chao K. Potential of surface-enhanced Raman spectroscopy for the rapid identification of Escherichia coli and Listeria monocytogenes cultures on silver colloidal nanoparticles. Appl Spectrosc. 2007;61(8):824-31.
28. Krafft C, Neudert L, Simat T, Salzer R. Near infrared Raman spectra of human brain lipids. Spectrochimica Acta Part A: Molecular and Biomolecular Spectroscopy. 2005;61(7):1529-35.
